# Patient engagement and problematic behaviours in nurse-staffed residential rehabilitation units

**DOI:** 10.1192/pb.bp.113.045252

**Published:** 2014-12

**Authors:** Alan Meaden, Martin Commander, Colin Cowan, Tom Edwards

**Affiliations:** 1 Birmingham and Solihull Mental Health NHS Foundation Trust; 2 St George’s Park, Morpeth; 3 Walsall Assertive Outreach and Rehabilitation Service, Dorothy Pattison Hospital

## Abstract

**Aims and method** To build on previous research findings by examining engagement and problematic behaviours of patients in 10 residential rehabilitation units. Two measures were completed on patients in community rehabilitation, longer-term complex care and high-dependency units (109 patients in total). Data were analysed and categorised into higher-engagement ratings across the domains of engagement and behaviour over the past 6 months and lifetime in terms of presence of the behaviour and likelihood of resulting harm.

**Results** Data were available for 73% of patients. All aspects of engagement were consistently low for all units, with highest levels in community rehabilitation units. Levels of problematic behaviours were similar across all units. Socially inappropriate behaviours and failure to complete everyday activities were evident for over half of all patients and higher for lifetime prevalence. Verbal aggression was at significantly lower levels in community units. Lifetime behaviours likely to lead to harm were much more evident in high-dependency units.

**Clinical implications** Despite some benefits of this type of care, patients continue to present challenges in engagement and problematic behaviours that require new approaches and a change in focus.

A significant minority of patients with severe and enduring mental illness do not engage well with psychiatric services. Indeed, the need to promote engagement was a key driver in the development of assertive outreach teams.^1^ However, it remains the case that even with the introduction of these intensive services there continue to be patients who fail to cooperate with community treatment plans and require admission to hospital.^2^ Little attention has thus far been paid to the concept of engagement in patients in specialist residential rehabilitation services, despite their stated aim of working with patients who have become ‘stuck’ in their recovery.^3,4^

This is all the more surprising given that engagement is a critical component in (positive) risk management.^5^ It is well recognised that patients not actively involved in their own treatment are more prone to be violent.^6^ The need for higher levels of support has been consistently demonstrated to be associated with problematic behaviours.^7^ There remains a group of longer-stay patients in both acute wards^8^ and in-patient rehabilitation settings who exhibit problematic behaviours that act as enduring barriers to successful community living.^3^

This study examines these two key factors, patient engagement and problematic behaviours, in patients on 24h nurse-staffed residential rehabilitation units.

## Method

A cross-sectional survey of patients in units provided by two National Health Service mental health trusts (covering a population of approximately 1.5 million across the three boroughs of Birmingham, Solihull and Sandwell within the West Midlands) was undertaken to examine care pathways and patient characteristics.^9^ The units were categorised as community rehabilitation, longer-term complex care and high-dependency, in accordance with the definitions adopted by the Royal College of Psychiatrists.^4^

The data from one trust (Birmingham and Solihull) were obtained (by A.M.) from the named nurse for each patient and supplemented with details from the case notes, and for the second trust the data were obtained (by C.C.) from senior nursing staff. A simple form was used to collect demographic and clinical data. In addition, two specific questionnaires were completed, the Residential Rehabilitation Engagement Scale^10^ and the Challenging Behaviour Checklist for Psychosis.^11^

The Residential Rehabilitation Engagement Scale is a 16-item staff-rated measure designed to capture engagement in in-patient rehabilitation settings during the past month. It consists of three dimensions: medication compliance; agreement with treatment and basic relationships; and active participation and openness. Each item is rated on a 5-point Likert scale, ranging from 1 (never) to 5 (always). The presentation of the engagement data was simplified by dichotomising them into usually/always *v*. the remainder.

The Challenging Behaviour Checklist for Psychosis is a 117-item measure of predefined problematic behaviours. Each type of behaviour is rated by staff in terms of the level of severity on a 0 to 4 scale (behaviour likely to result in social exclusion through to ability to cause serious harm or death) and frequency/recency on a 0 to 8 scale (ranging from lifetime occurrence to daily incidents). Items are categorised into 11 domains. Data relating to the past 6 months and lifetime are presented for all behaviours and then separately for problematic behaviours leading to imminent physical harm to self or others.

The data are presented descriptively. Chi-squared tests were used to compare engagement and problematic behaviours across the three types of rehabilitation unit. Data were analysed using SPSS 12.0.1 for Windows.

The project did not require ethical committee approval (as determined by the National Research Ethics Service), but agreement was obtained from the relevant clinical governance bodies in both trusts.

## Results

Of the 10 units included, 5 were classified as community rehabilitation units (44 patients), 3 as complex care units (36 patients) and 2 as high-dependency units (29 patients). Demographic and clinical data were available for 98/109 (90%) patients. Sixty-six patients were men and their mean age was 45 years. Sixty-six patients were White, 22 were Black/Black-mixed and 10 were from other ethnic groups. None of the patients were in employment and only 5 were currently married. Sixty-six patients had a diagnosis of schizophrenia, 26 had affective psychoses (depressive and bipolar) and 5 had other diagnoses; for 1 patient no diagnosis was available. The median length of stay of these

**Table 1:** Engagement

Engagement domain/items (rated as ‘usually’ or ‘always’)	Total (*n* = 88)	Community (*n* = 30)	Complex care (*n* = 30)	High dependency (*n* = 28)	χ^2^	*P*
Adheres to medication, *n* (%)	72 (83)	25 (86)	24 (83)	23 (82)	-	ns
						
Basic relationships, *n* (%)						
Relates well with the team	44 (50)	20 (67)	13 (43)	11 (39)	-	ns
Relates well with named nurse	61 (69)	22 (73)	21 (70)	18 (64)	-	ns
Relates well with therapy staff	42 (48)	17 (57)	14 (47)	11 (39)	ns
Discuss and agree with proposed intervention	33 (38)	17 (57)	9 (30)	7 (25)	7.29	0.03
Involved in proposed intervention if prompted	34 (39)	18 (60)	10 (33)	6 (21)	9.63	0.008
						
Active participation, *n* (%)						
Discusses personal feelings	26 (30)	12 (40)	9 (30)	5 (18)	-	ns
Discusses personal problems	24 (27)	11 (37)	8 (27)	5 (18)	-	ns
Discusses symptoms	18 (21)	6 (20)	5 (17)	7 (25)	-	ns
Discusses behaviours	15 (17)	7 (23)	5 (17)	3 (11)	-	ns
Identifies realistic goals	9 (10)	6 (20)	1 (3)	2 (7)	-	ns
Perceives rehabilitation as useful	16 (18)	11 (37)	2 (7)	3 (11)	10.61	0.005
Actively involved in proposed intervention	20 (23)	9 (30)	6 (20)	5 (18)	-	ns
Driven by perceived benefit of intervention	15 (17)	10 (33)	4 (14)	1 (4)	9.35	0.009
Completes rehabilitation goals	15 (17)	8 (28)	4 (13)	3 (11)	-	ns
Keeps appointments without support	25 (29)	12 (41)	8 (27)	5 (18)	-	ns

ns, non-significant.

patients was 38 months (range 0–143; see Cowan *et al*^9^ for further details).

Data on engagement were available for 88/109 (73%) patients (Table 1). The engagement scores were consistently low across all types of unit; those in the domain of ‘active participation’ were especially so. Only two items were rated (usually/always) greater than 50%, namely ‘relates well with named nurse’ and ‘complies with medication’. There were significant differences between the units on 4 of the 16 variables; greater levels of engagement were reported for patients in community rehabilitation than in complex care or high-dependency units.

Data on problematic behaviours were obtained for 80/109 (73%) patients (Table 2). Over half of the patients continued to present with verbal aggression, showed socially inappropriate behaviours (e.g. making excessive demands/complaints or urinating in public), and failed to complete everyday activities (e.g. refused to wash/dress appropriately or stayed in bed/their room); the last two items were significantly more common in patients on complex care units. Problematic behaviours likely to result in imminent physical harm to self or others were uncommon aside from ‘failure to perform a range of everyday activities’ (35%). Furthermore, there were no significant differences in these items over the previous 6 months across the three types of unit.

By contrast, lifetime prevalence of problematic behaviours was uniformly high, with the exception that absconding was significantly lower for those in complex care units, as were levels of verbal aggression for patients in community rehabilitation units (Table 3). Also notable were the very high levels across all units on ‘failure to perform a range of everyday activities’, second only to verbal aggression. The ratings for problematic behaviours leading to imminent physical harm to self or others were evident to a much greater degree across the board for patients in high-dependency units; in most instances these findings were statistically significant.

**Table 2:** Problematic behaviours: 6 months

Any behaviour	Total (*n* = 80)	Community (*n* = 29)	Complex (*n* = 25)	High-dependency (*n* = 26)	χ^2^	*P*
Self-harm, *n* (%)	12 (15)	2 (7)	4 (16)	6 (23)	-	ns
						
Verbal aggression, *n* (%)	44 (55)	13 (45)	16 (64)	15 (58)	-	ns
						
Physical aggression against objects, *n* (%)	11 (14)	3 (10)	3 (12)	5 (19)	-	ns
						
Physical aggression towards others, *n* (%)	27 (34)	8 (28)	11 (44)	8 (31)	-	ns
						
Sexually Inappropriate behaviours, *n* (%)	27 (34)	7 (24)	10 (40)	10 (39)	-	ns
						
Fire risk behaviours, *n* (%)	14 (18)	7 (24)	5 (20)	2 (8)	-	ns
						
Compulsive behaviours, *n* (%)	33 (41)	11 (38)	12 (48)	10 (39)	-	ns
						
Acquisitive behaviours, *n* (%)	5 (6)	2 (7)	1 (4)	2 (8)	-	ns
						
Absconding, *n* (%)	9 (24)	9 (31)	7 (28)	3 (12)	-	ns
						
Socially inappropriate behaviours, *n* (%)	57 (71)	20 (69)	22 (88)	15 (58)	5.831	0.054
						
Failure to perform range of everyday activities, *n* (%)	59 (74)	23 (79)	22 (88)	14 (54)	8.406	0.015
						
Behaviour leading to imminent physical harm to self or others, *n* (%)						
Self-harm	3 (4)	1 (3)	0 (0)	2 (8)	-	ns
Verbal aggression	10 (13)	1 (3)	3 (12)	6 (23)	-	ns
Physical aggression against objects	6 (8)	1 (3)	2 (8)	3 (12)	-	ns
Physical aggression towards others	14 (18)	2 (7)	4 (16)	8 (31)	-	ns
Sexually inappropriate behaviours	6 (8)	0 (0)	3 (12)	3 (12)	-	ns
Fire risk behaviours	10 (13)	4 (14)	4 (10)	2 (8)	-	ns
Compulsive behaviours	7 (9)	1 (3)	3 (12)	3 (12)	-	ns
Acquisitive behaviours	0 (0)	0 (0)	0 (0)	0 (0)	-	-
Absconding	9 (11)	3 (10)	4 (16)	2 (8)	-	ns
Socially inappropriate behaviours	8 (10)	0 (0)	3 (12)	5 (19)	-	ns
Failure to perform range of everyday activities	28 (35)	7 (24)	12 (48)	9 (35)	-	ns

ns, non-significant.

## Discussion

The poor level of engagement reported for patients in all three types of rehabilitation unit was striking. The scores for community units, as might have been expected, showed a higher level of active participation than either complex care or high-dependency units, although it should be acknowledged that this applied to only around a third of patients. Even at the basic level of discussing and agreeing with proposed interventions, only around two-thirds participated; this gives an indication of the challenge facing units whose key purpose is to promote recovery and assist patients in moving on to more independent accommodation. By comparison, in a community sample of patients under an assertive outreach team in Birmingham only a quarter were classified as poorly engaged.^1^ This reinforces a view that there is a core group of patients who, despite the best efforts of community services, remain stubbornly disengaged and may require a longer period of in-patient treatment.^2^

The uniformly disappointing commitment to rehabilitation interventions is frustrating given that many patients in all three types of unit related well to their named nurse. This is possibly because these relationships serve to meet more immediate needs whereas rehabilitation programmes often target medium- to longer-term goals, which may be perceived by patients as an irrelevance or imposition. Relating well to one individual may also detract from relating well to the whole team. The challenge is to promote engagement with the whole team as well as broader rehabilitation process, perhaps by building other interventions on the rapport established between patients and their named nurses. Systemically informed treatments that seek to promote an open and shared dialogue regarding problems, their causes and treatment with the whole treatment team, patient and carers may prove effective in promoting better involvement in care-planning and goal-setting.^12^

The lack of therapeutic engagement is of particular concern, given the considerable problematic behaviours shown by these patients, not only with respect to behaviours required for independent living (‘failure to perform a range of everyday activities’), but also the persistently high levels of verbal aggression and socially inappropriate behaviours. Alongside other issues, such as the availability of suitable community alternatives,^3^ this in part explains why patients in this sample had not yet moved on.^9^ A comparison of the lifetime and 6-month data suggest that problematic behaviours may have reduced, pointing to the benefit of these services (especially those in high-dependency units) in providing the necessary supervision and containment to manage risks. However, a lack of willingness to discuss symptoms and behaviours makes it likely that patients may well not have grasped the factors driving problematic behaviours. In the absence of ongoing rehabilitation interventions therefore it is probable that any difficulties would simply re-emerge given time.

This raises the question as to how we can work more effectively with these patients to enable them to successfully move on to less supervised settings, possibly

**Table 3:** Problematic behaviours: lifetime

Any behaviour	Total (*n* = 80)	Community (*n* = 29)	Complex care (*n* = 25)	High-dependency (*n* = 26)	χ^2^	*P*
Self-harm, *n* (%)	36 (45)	12 (41)	9 (36)	15 (58)	-	ns
						
Verbal aggression, *n* (%)	64 (80)	17 (57)	22 (88)	25 (96)	13.525	0.001
						
Physical aggression against objects, *n* (%)	31 (39)	9 (31)	6 (24)	16 (62)	8.708	0.013
						
Physical aggression towards others, *n* (%)	51 (64)	16 (55)	14 (56)	21 (81)	-	ns
						
Sexually Inappropriate behaviours, *n* (%)	44 (55)	13 (45)	14 (56)	17 (65)	-	ns
						
Fire risk behaviours, *n* (%)	28 (35)	9 (31)	9 (36)	10 (39)	-	ns
						
Compulsive behaviours, *n* (%)	39 (49)	13 (45)	14 (56)	12 (46)	-	ns
						
Acquisitive behaviours, *n* (%)	17 (21)	9 (31)	2 (8)	6 (23)	-	ns
						
Absconding, *n* (%)	48 (60)	19 (66)	10 (40)	19 (73)	6.387	0.041
						
Socially inappropriate behaviours, *n* (%)	71 (89)	24 (83)	23 (92)	24 (92)	-	ns
						
Failure to perform range of everyday activities, *n* (%)	76 (95)	26 (90)	25 (100)	25 (96)	-	ns
						
Behaviour leading to imminent physical harm to self or others, *n* (%)						
Self-harm	25 (31)	8 (28)	5 (20)	12 (46)	-	ns
Verbal aggression	31 (39)	5 (17)	9 (36)	17 (65)	13.503	0.001
Physical aggression against objects	20 (25)	2 (7)	3 (12)	15 (58)	22.143	0.000
Physical aggression towards others	34 (43)	7 (24)	7 (28)	20 (77)	18.759	0.000
Sexually inappropriate behaviours	18 (23)	1 (3)	5 (20)	12 (46)	14.468	0.001
Fire risk behaviours	19 (24)	5 (17)	5 (20)	9 (35)	-	ns
Compulsive behaviours	8 (10)	1 (3)	3 (12)	4 (15)	-	ns
Acquisitive behaviours	2 (3)	0 (0)	0 (0)	2 (8)	-	ns
Absconding	28 (35)	5 (17)	6 (24)	17 (65)	15.901	0.000
Socially inappropriate behaviours	18 (23)	0 (0)	3 (12)	15 (58)	28.467	0.000
Failure to perform range of everyday activities	45 (56)	9 (31)	14 (56)	22 (85)	15.994	0.000

ns, non-significant.

in the community. There remains a role for standard cognitive–behavioural therapy (CBT) for treatment-resistant patients as well as other psychological interventions for those patients who are better able to discuss their experiences and to actively become involved in their treatment. However, for many this is unlikely to be successful given persistent problems with engagement which is needed as a basis for active collaborative therapies such as CBT. One alternative is to utilise interventions that explicitly focus on treatment-resistant behaviours as barriers to personal recovery. This shifts interventions away from symptoms or disabilities and redirects the goal towards engagement and reducing problematic behaviours. The Challenging Behaviour Checklist^11^ items provide a useful framework around which it is possible to construct intensive team-based interventions not otherwise possible in the community. More intensive psychological input, along with staff training focused on addressing problem behaviours for difficult-to-place patients in a systematic way, has been shown to be effective in reducing aggressive and problematic social behaviour and removing barriers to resettlement into the community.^13^ Behavioural approaches continue to hold value for this group, alongside team-based cognitive therapy,^11^ to provide more frequent and more timely interventions linked to warning signs for problem behaviours while addressing any unhelpful team attitudes towards patients and their behaviour. A functional analysis of problem behaviours and poor engagement should aim to ensure that contextual and non-illness factors are also addressed (e.g. social skills, personal attitudes). Engagement with the named nurse is clearly crucial and should provide the platform for such interventions with the fuller multidisciplinary team. Training should ensure that named nurses in these units are fully equipped to deliver these interventions where engagement with other professionals is not possible.

For some patients, even these efforts may prove insufficient to promote recovery and maintain them in community living. Such individuals may require longer-term complex care facilities that are able to provide the necessary supervision and monitoring required for safely managing risk while continuing to promote and maximise quality of life. There is clearly a strong case for the continued existence of these units in a spectrum of mental health provision.^3^

The limited progress to greater independence observed in this group^9^ does not simply reflect a more severe form of treatment-resistant illness but also the consequences of poor engagement and problem behaviours. Routinely assessing engagement and problematic behaviours, alongside the success of any interventions, may usefully help to determine the need for longer-term intensive support or potential for discharge. The ability of a wider network of providers to appropriately support such individuals after discharge needs to be considered.

## References

[1] Meaden A Nithsdale V Rose C Smith J Jones C Engagement and other factors associated with outcome in assertive outreach. J Ment Health 2004; 13: 415–24.

[2] Commander M Sashidharan S Rana T Ratnayake T North Birmingham assertive outreach evaluation: patient characteristics and clinical outcomes. Soc Psychiatry Psychiatr Epidemiol 2005; 40: 988–93. 16341614 10.1007/s00127-005-0989-8

[3] Holloway F The Forgotten Need for Rehabilitation in Contemporary Mental Health Services (Faculty Report FR/RS/04). Royal College of Psychiatrists’ Faculty of Rehabilitation and Social Psychiatry, 2005 (http://www.rcpsych.ac.uk/pdf/FR_RS_04.pdf).

[4] Wolfson P Holloway F Killaspy H (eds) Enabling Recovery for People with Complex Mental Health Needs: A Template for Rehabilitation Services (Faculty Report FR/RS/1). Royal College of Psychiatrists’ Faculty of Rehabilitation and Social Psychiatry, 2009 (http://www.rcpsych.ac.uk/pdf/fr_rs_1_forwebsite.pdf).

[5] Department of Health. Best Practice in Managing Risk: Principles and Evidence for Best Practice in the Assessment and Management of Risk to Self and Others in Mental Health Services. Department of Health, 2007.

[6] Monahan J Steadman HJ Silver E Appelbaum PS Robbins PC Mulvey EP Rethinking Risk Assessment: The MacArthur Study of Mental Disorder and Violence. Oxford University Press, 2001.

[7] Mulholland C Wilson C McCrum B MacFlynn G Factors influencing the support offered to the seriously mentally ill by a community rehabilitation service. J Ment Health 2009; 8: 639–47.

[8] Commander C Rooprai D Survey of long-stay patients on acute psychiatric wards. Psychiatr Bull 2008; 32: 380–3.

[9] Cowan C Meaden A Commander C Edwards T In-patient psychiatric rehabilitation services: survey of service users in three metropolitan boroughs. Psychiatrist 2012; 36: 85–9.

[10] Meaden A Hacker D de Villiers A Carbourne J Paget A Developing a measurement of engagement: The Residential Rehabilitation Engagement Scale for psychosis. J Ment Health 2012; 21: 182–91. 22559829 10.3109/09638237.2012.664301

[11] Meaden A Hacker D Problematic and Risk Behaviours in Psychosis: A Shared Formulation Approach. Routledge, 2011.

[12] Seikkula J Alakare B Aaltonen J Holma J Rasinkangas A Lehtinen V Open dialogue approach: treatment principles and preliminary results of a two-year follow-up on first episode schizophrenia. Ethical Hum Sci Serv 2003; 5: 163–82.

[13] Leff J Szmidla A Evaluation of a special rehabilitation programme for patients who are difficult to place. Soc Psychiatry Psychiatr Epidemiol 2002; 37: 532–6. 12395143 10.1007/s00127-002-0578-z

